# Response of soil bacterial populations to application of biosolids under short-term flooding

**DOI:** 10.1007/s11356-023-27424-0

**Published:** 2023-05-15

**Authors:** Nicholas H. Humphries, Steven F. Thornton, Xiaohui Chen, Andrew W. Bray, Douglas I. Stewart

**Affiliations:** 1 School of Civil Engineering, University of Leeds, Leeds, LS2 9JT UK; 2 Currently Anglo American plc, 17 Charterhouse St, London, EC1N 6RA UK; 3 Department of Civil and Structural Engineering, University of Sheffield, S1 3JD, Sheffield, UK; 4 School of Earth and Environment, University of Leeds, Leeds, LS2 9JT UK; 5 Currently Calder Rivers Trust, Halifax, HX1 5ER UK

**Keywords:** *Acidobacteria*, Agriculture, Biosolids, *Firmicutes*, Flooding, Microbiology, *Proteobacteria*, Soil

## Abstract

Biosolids are applied to agricultural land as a soil conditioner and source of crop nutrients. However, there is concern that bacteria from biosolids may become established in soils, particularly if that soil becomes water-logged. This study examined the microbial community of arable soils cultivated with barley under different applications of biosolids (0, 24t/ha, 48t/ha) in laboratory mesocosms which simulated a 10-day flood. Nutrients (P and N) and organic matter in the soil increased with application rate, but plant growth was not affected by biosolid application. The biosolids contained 10× more genetic material than the soil, with much lower bacterial diversity, yet application did not significantly change the taxonomy of the soil microbiome, with minor changes related to increased nutrients and SOM. Anaerobic conditions developed rapidly during flooding, causing shifts in the native soil microbiome. Some bacterial taxa that were highly abundant in biosolids had slightly increased relative abundance in amended soils during the flood. After flooding, soil bacterial populations returned to their pre-flood profiles, implying that the native microbial community is resilient to transient changes. The short-term changes in the microbiome of biosolid-amended soils during flooding do not appear to increase the environmental risk posed by biosolid application.

**Supplementary Information:**

The online version contains supplementary material available at 10.1007/s11356-023-27424-0.

## Introduction

Digested sewage sludge (biosolids) is becoming increasingly popular for application to agricultural land and recognised as a valuable soil conditioner and nutrient source. Historically there have been concerns about the practice due to the potential transfer of pathogenic microorganisms to soil and the accumulation of harmful chemicals (Sterritt and Lester 1980; Pepper et al. 2006). However, many improvements have been made in the use of biosolids in agriculture due to legislation and assurance schemes at both EU and local (UK) level. These include, but are not limited to, the European Council Directive 86/278/EEC ‘Sewage Sludge Directive’ (The Council of European Communities 1986), ‘The Sludge (Use in Agriculture) Regulations 1989’ (Department of the Environment 1989), and the ‘Biosolids Assurance Scheme’ (Assured Biosolids Limited 2018). Additionally, improved wastewater practices that reduce chemical contaminants disposed to sewer and advanced digestion processes that kill pathogens mean that biosolid application is now considered a safe and sustainable alternative to inorganic fertilisers (Smith 2009; Clarke and Smith 2011; European Commission 2014; Al-Gheethi et al. 2018).

The value of biosolids arises mainly from its high nitrogen (N) and phosphorus (P) content. The N contained in biosolids can reduce the need for expensive chemical N fertiliser, which has a high energy cost for its production (Basosi et al. 2014). The high P content in biosolids may also reduce dependency on the diminishing global stock of mineral P (Torri et al. 2017). Furthermore, biosolids are a source of organic carbon (C) and increase soil organic matter (SOM) which is a key indicator of soil fertility and can lead to higher attainable crop yields (Singh and Agrawal 2008; Johnston et al. 2009; Hijbeek et al. 2017).

Climate change has implications for the management of agricultural soils. Changes in annual rainfall patterns and an increased number of high intensity rainfall events have been documented in Europe in recent years, including major floods in the UK in 2012, 2014, and 2019 (Met Office 2019). Such changes are predicted to continue in the future (Lenderink and van Meijgaard 2008; Falloon and Betts 2010; Kendon et al. 2014; Centre for Ecology & Hydrology 2016). Waterlogging of low-lying and heavy soils, flash-flooding of fields, and floodplain storage of water as an urban flood alleviation measure are all potential outcomes of extreme rainfall events (Wheater and Evans 2009). Thus, maintenance of soil health and quality to mitigate damage to crops and soils from flooding is essential land management practice (Kiss et al. 2021a; Kiss et al. 2021b). One method of improving soil structure is to increase SOM with organic C inputs such as manures, straw, composts, and biosolids (Haynes and Naidu 1998; Masri and Ryan 2006).

Biosolids are created after sewage sludge undergoes dewatering and some form of secondary treatment, which can include aerobic digestion, anaerobic digestion (AD), composting, or lime stabilisation. The aim of these digestion processes is to break down organic matter and to raise the temperature of the biosolid to eliminate pathogenic organisms that may have been present in the wastewater (Mello Leite Moretti et al. 2015; Al-Gheethi et al. 2018). Additionally, pretreatment steps can be included prior to digestion, such as thermal hydrolysis which uses high temperatures and pressure to improve organic matter breakdown and pathogen control (Gahlot et al. 2022). In the UK 73% of biosolids are produced using AD (Assured Biosolids Limited 2018). AD degrades wastewater sludge in a controlled environment to produce biogas and to reduce GHG emissions, as well as effectively control pathogens (DEFRA 2011).

Despite effective control of pathogenic microbes in biosolids from secondary treatment, there may still be a low risk that some microorganisms, either pathogens or anaerobes from the AD process itself, may persist in soils under specific environmental conditions (Chen et al. 2011; Zhao and Liu 2019). Fresh biosolids contain anaerobic bacteria from the AD process and are an abundant source of nutrients and organic C (Kathijotes et al. 2016). Therefore, applying AD-derived biosolids to soils, which become anaerobic under flooded conditions, could provide an environment in which some anaerobes contained in the biosolids outcompete the native soil microbiome. This could cause unforeseen negative impacts on the function of the native soil microbiome and may then spread to watercourses and the wider environment causing potential damage to native ecosystems.

This study investigates the impact of biosolid application and a short-term flood event on soil bacterial population composition and diversity. It assesses whether anaerobic bacteria which are characteristic of anaerobic digestion survive in agricultural soil under cropping and whether a temporary anoxia induced by short-term flooding results in a resurgence in their number. Mesocosm experiments were constructed to simulate arable field growth conditions in a controlled environment to allow focus on the effects of biosolid application rate without interference from variable environmental conditions. Amplicon sequencing data from the V4 hypervariable region of the 16s rRNA gene at different timepoints was then used to characterise soil bacterial populations in response to a 10-day stagnant flood event.

## Materials and methods

### Soil and biosolids

A representative sample of topsoil was collected from a working arable field at Spen Farm, Tadcaster, UK (53.8699, −1.3290) in mid-October 2018. The field was newly established with wheat at the time of soil collection, so soil samples were taken between plants from 20+ randomly selected locations across the field using a Dutch auger. The soil was characterised as a Cambisol, with a medium-strong silt loam texture containing some stone fragments derived from the underlying calcareous mudstone and dolomitic limestone (Harrogate Till formation) (Jarvis et al. 1984). The field had received an application of triple-superphosphate at drilling in early August, corresponding to a 64kg/ha P application, with no other fertiliser applications since drilling. A sample of the soil was analysed at a commercial laboratory and found to have no nutrient deficiencies or excesses which would potentially affect crop growth. The collected soil was preserved in a field-moist condition without sieving, to minimise disturbance of its microstructure and to preserve microbial communities.

Biosolids were sourced from Esholt Water Treatment Works in West Yorkshire, UK. The biosolid was a digested sewage sludge cake derived from secondary treatment of municipal wastewater that had undergone thermal hydrolysis at a temperature of 165°C and a pressure of 6 bar for a period of 30 min prior to AD. An initial biosolid sample was characterised before the experiment, and the results are shown in Table S1.

### Mesocosm setup

Six growth box mesocosms were constructed using clear plastic boxes with an area of 0.165m^2^ (50 × 33 cm) and a depth of 30cm. The boxes were spray-painted black to a height of 18cm to prevent light infiltration into the soil layer but permit viewing of the floodwater level above the soil surface. A 3-cm gravel layer that could be drained by an external valve in the bottom of each box was created to simulate an agricultural field drain. Each box was filled with 34kg of soil to create a 15-cm soil layer over the drainage layer with a wet soil bulk density of 1.37g/cm^3^.

Mesocosms were set up with a three different soil conditions: a control with no biosolid applied, a typical biosolid application rate (400g biosolid, approximately 24t/ha), and a high biosolid application rate (800g biosolid, approximately 48t/ha). The biosolid was mixed into the whole soil layer by hand to simulate a field cultivation, and control soils were mixed in the same way. After mixing the soil, 60 barley (*Hordeum vulgare*) seeds were planted at a depth of 2cm in three evenly spaced rows of 20. A second set of mesocosms were set up using the same parameters.

The ‘typical’ biosolid application rate was intended to reflect actual agricultural practice in the region, which usually conforms with EU guidance for a maximum field application rate of 250kg N ha^−1^ in a nitrate vulnerable zone (NVZ) (DEFRA 2013). The ‘high’ rate represents an extreme condition of twice the maximum application. Biosolids were applied as soon as practicable after collection as they would be on-farm. This was before the analytical results were available, so nutrient values for a standard ‘digested cake’ from RB209: Nutrient Management Guide (AHDB 2017) were used to determine the application rates. As a result, the ‘typical’ application rate exceeds the UK national guidance for an NVZ by 30% (corresponding to 345kg N ha^−1^), indicating the batch variability of digested sewage cake.

The two sets of three mesocosms were placed in two growth chambers in randomised order. The growth chambers consisted of a steel frame covered with Mylar foil and two LED growth lamps (54W LED grow lamps, Model: HY-55cm-18*3W-RB, UK) mounted at the top of the frame, powered with timer switches on a 12-h on/off cycle. The lamps delivered 65.3–80.4 μmol m^−2^s^−1^ of photosynthetically active radiation in the 400–700-nm range, measured at the level of the soil surface. The barley crop was established in the mesocosms over 28 days. During this growth period, the mesocosms were surface watered twice per week to a point when the bottom drain on each box started flowing, at which point the drain tap was closed. Any drained water was then re-added to the mesocosm to preserve its nutrient content. This watering regime was intended to simulate field capacity in the soils, avoiding waterlogging but allowing for between-box variation in soil water holding capacity.

### Flood simulation

After 28 days of crop growth, a 10-day flood was initiated by inundating the mesocosms with water to a depth of 5cm above the soil surface. The water level was maintained throughout the flood by carefully topping it up using a syringe to minimise disturbance and maintain stagnant flood conditions.

Measurements of dissolved oxygen (DO), pH, and oxidation-reduction potential (ORP) were made daily in the surface water and at a depth of 5cm and 10cm below the soil surface. The pH and DO of the surface water were measured with dipping probes carefully inserted into the water to minimise disturbance (Hach Intellical™ LDO101-30 and Hach Intellical™ LDO101-30 used with a HQ40D portable multimeter, Hach UK). Porewater pH was measured by inserting the probe into standpipes permanently installed at a depth of 5cm and 10cm. Porewater DO was measured using oxygen sensor “spots” (PreSens Oxygen Sensor Spot SP-PSt3-NAU, PreSens, Germany). These were fixed to the underside of clear plastic tubes permanently inserted into the soil. Measurements were taken using a fibre optic detector after calibration following the manufacturer’s instructions.

ORP was measured using Pt electrodes constructed as per the method outlined by Farrell et al. (1991). The Pt electrodes were placed in the surface water and permanently mounted in the mesocosms at depths of 5cm and 10cm below the soil. Measurements were taken using a voltmeter (TENMA environmental multimeter, P/N IN05691, UK) connected between the Pt electrodes and an AgCl reference electrode (Sentek pH electrode, P/N P11-DJ, UK) which was inserted into the surface water and allowing to equilibrate for 2 min.

Upon completion of the 10-day flood, the floodwater was drained from the valve at the bottom of each mesocosm. The mesocosms were then allowed 20 days for flood recovery (2× flood duration), under the same conditions as the initial growth.

### Sampling

Before starting the experiment, three samples (~500g each) of both the initial soil and fresh biosolid were collected, frozen (−20°C), and stored for later preparation and chemical analysis. Five samples of each material (~2g each) were also collected using a sterile spatula and frozen (−20°C) for bacterial DNA extraction.

During the experiment, soil samples were taken from each mesocosm for chemical and microbial analysis at three separate timepoints: immediately before flooding (pre-flood), immediately after flood draining (post-flood), and at the end of the 20-day recovery period when terminating the experiment (final). At each timepoint, three soil samples were taken from each mesocosm in a randomised pattern using a 2.5-cm diameter core auger to a depth of 15cm and frozen (−20°C) for later analysis. The insides of each hole left by the core auger were then scraped with a disposable sterile inoculation loop to collect ~2g of soil from the whole depth of the soil profile. These samples were also stored at −20°C for bacterial DNA extraction. The holes left by the core auger were then backfilled with loose soil from the soil surface.

### Soil chemical analysis

When all samples had been taken, the bulk soil samples were thawed and air dried for 48 h, disaggregated, and allowed to air dry again to reach a constant mass. Each sample was weighed and ground to pass through a 2-mm sieve, with any material not passing through weighed and discarded. The remaining material was then all ground to pass through a 355-μm sieve to improve sample uniformity and amalgamation. SOM was then determined by the Walkley-Black dichromate method (Walkley and Black 1934). Plant available P was determined using Olsen’s reagent (Olsen et al. 1954) and measured colorimetrically in solution by spectrophotometry using the molybdenum blue method (Murphy and Riley 1962). Soil N content was determined as Total Kjeldahl Nitrogen (TKN) using the method 4500-N_org_ Nitrogen (Organic) (Standard Methods for the Examination of Water and Wastewater 2018) with a 50:1 dilution of water/soil, copper catalyst tablets (Fisher Scientific, 1g Na_2_SO_4_ and the equivalent of 0.1g CuSO_4_, Fisher chemical K/0120/80), a Buchi B-435 Digestion Unit, and Buchi B324 Distillation Unit. pH was measured using a 1:2.5 v/v suspension in water (World Agroforestry Centre 2014) using a Hach Intellical PHC201 pH electrode and Hach HQ40D portable multimeter.

### Bacterial characterisation

Microbial genomic DNA was extracted from subsamples (~0.25 g) taken from the small 2g soil and biosolid samples using a DNeasy PowerSoil Kit (QIAGEN Ltd,). DNA fragments in the size range of 3~20kb were isolated by electrophoresis using a 1% agarose 1x Tris-borate-EDTA (TBE) gel stained with ethidium bromide (EtBr) for viewing under UV light. DNA was extracted from excised gel fragments using a QIAquick gel extraction kit (QIAGEN Ltd), with final elution carried out using a 1/10th strength elution buffer. DNA concentration was quantified using a Qubit™ dsDNA HS Assay Kit on a Qubit® 2.0 Fluorometer (Invitrogen). The manufacturer’s instructions for each kit were followed precisely unless otherwise stated.

A 20-μl volume of the extracted DNA samples in aqueous solutions ranging in concentration from 0.8 to 35.4 ng/μl were analysed at the University of Liverpool Centre for Genomic Research (CGR) for paired-end (2×250bp) sequencing on the Illumina MiSeq platform. Illumina adapters and barcodes were attached to the DNA fragments in a 2-step PCR amplification to target the hyper-variable V4 region of the 16s rRNA gene of bacteria as per Caporaso et al. (2011). The target-specific part of the primer sequences used were the 515F (FWD:GTGYCAGCMGCCGCGGTAA) (Parada et al. 2016) and 806R (REV:GGACTACNVGGGTWTCTAAT) (Apprill et al. 2015).

### Data processing

After sequencing, the raw reads were trimmed by the CGR to remove the Illumina adapter sequences using Cutadapt version 1.2.1 (Martin 2011). The option -O 3 was used, wherein the 3′ end of any reads which match the adapter sequence for 3 bp or more were trimmed. The reads were further trimmed using Sickle version 1.200 (Joshi and Fass 2011) with a minimum window quality score of 20. Reads shorter than 20bp after trimming were removed.

The trimmed reads were processed using the UPARSE pipeline (Edgar 2013) within the USEARCH software package (version 11) (Edgar 2010). First, paired-end reads were assembled using the *fastq_mergepairs* command. Next, the target-specific primer sequences were stripped, and then the paired-end reads were truncated to 250bp, and reads shorter than 250bp were discarded using the *fastx_truncate* command. The outcome was that any partial reads were discarded, and any longer, poorly merged reads were shortened to the target 250bp of the V4 region of the 16s rRNA. The low quartile, median, and high quartile lengths of the untruncated reads were all 253bp. However, 250bp was selected as the length of truncation to preserve reads which may have been missing only a few base pairs but were otherwise valid. Only 0.2% of reads were discarded due to insufficient length. After truncating, the reads were quality filtered using the *fastq_filter* command, with an expected error of 1.0. Samples were then de-replicated and relabelled using the *fastx_uniques* command, and all reads were pooled. Clustering and chimera filtering of reads was then carried out simultaneously using the *cluster_otus* command, with a minimum abundance of 2 reads used to eliminate singletons and a sequence identity threshold of 97% used to define operational taxonomic units (OTUs) (>96% passed the filtering stage).

An OTU table was generated using the *otutab* command, mapping the filtered reads to the OTUs. The discarded reads from truncating and filtering were assumed to be low quality or misreads and were not mapped to the OTU table. The *sintax* command with a *-sintax_cutoff* option set to 0.8, for a confidence cut off of 80%, was then used within the VSEARCH software package (Rognes et al. 2016) to process the SILVA 16s rRNA database v123 and assign taxonomies. Greater than 99% of OTUs were successfully assigned. Any OTUs which did not have a confidence value of at least 0.7 at bacterial phylum level or any which were classified as *Archaea* were discarded and not included in the diversity or statistical analysis.

### Statistical analysis

Hill numbers (D_q_) were used to determine bacterial diversity within all samples (Hill 1973). Hill numbers allow for the proportional representation of diversity within samples by weighting taxa by abundance. This method compensates for differences in sample size and accounts for rare taxa by differentially weighing them, therefore allowing comparison of diversity between samples. Hill numbers are now the preferred measures of bacterial diversity because their units are number of taxa, and proportional changes in these indices directly reflect changes in diversity (Roswell et al. 2021). The OTU richness (D_0_), common OTUs (D_1_, equivalent to the exponential of Shannon entropy), and dominant OTUs (D_2_, equivalent to the inverse of Simpson concentration) were used to characterise the samples. The approach of using Hill numbers has been shown to be a reliable estimation of diversity for next-generation sequenced bacterial communities (Chao et al. 2010; Kang et al. 2016). The USEARCH command *alpha_div* was used to calculate alpha diversity metrics for all samples.

Beta diversity metrics were obtained using the *beta_div* command in USEARCH to determine the relative differences between individual samples. The Bray-Curtis dissimilarity scores gained from this analysis showed biosolid samples to be highly dissimilar to all soil samples (>0.966, where 0 indicates that all OTUs are shared and 1 indicates that samples did not share any OTUs). Therefore, biosolid samples were excluded from further statistical analysis for comparison with soil samples. This preserved the differences between soil samples and allowed observation of effects caused by biosolid application or flooding more clearly. The OTU table of the 59 soil samples was input as a matrix to RStudio (version 1.2.5001) (RStudio Team 2019), and a non-metric multidimensional scaling (NMDS) analysis was carried out using the ‘vegan’ package (Oksanen et al. 2013) to graphically represent the dissimilarity of bacterial samples in a 2-dimensional space using the pairwise Bray-Curtis distances.

To investigate the relationship between soil properties and microbial communities, Spearman’s rank correlation was carried out on soil phyla abundances and the values of soil TKN, Olsen P, SOM, and pH from all soils. The soil in each box was randomly sampled for geochemical and microbial analysis, so mean values of each factor from the box were used rather than individual sample comparisons.

## Results

### Plant growth

Plant establishment after 1 week of growth was very low in the control tests (20 and 16 plants from 60 seeds), low in the typical rate tests (29 and 30 plants), and moderate in high-rate tests (41 and 48 plants). Extra seeds were added to all the mesocosms in proportion to the failure to establish, to produce similar plant numbers. After 28 days, the control tests contained 28 and 27 plants, the typical rate tests contained 49 and 42 plants, and the high-rate tests contained 53 and 52 plants. After flooding, there were plant deaths in most mesocosms (29% ± 16% plant death rate), but no trend related to biosolid application was observed. The dry weight biomass (DWB) of the plants in each mesocosm was determined after the experiment. There were no differences between the control and typical rate tests (0.18–0.23g mean per-plant DWB), but DWB was higher in high-rate tests (0.25g and 0.28g mean per-plant DWB). Flooding occurred during crucial early plant growth stages, and the experiment was terminated before plants reached maturity. Therefore, inferences should not be made about the impact of biosolids on plant growth from this experiment.

### Soil nutrient analysis

The Olsen P, TKN, and SOM contents of the soils all increased with the amount of biosolids applied but showed no systematic variation during the tests (Table 1). The Olsen P concentration in the initial soil was 19.8 ±3.3 mg P kg^−1^, but after 28 days of plant growth, it was 28.7 ±3.7 mg P kg^−1^ in the control boxes, and this increased to 38.8 ±3.4 and 39.9 ±5.0 mg P kg^−1^ with the typical and high biosolid application rates, respectively (data is reported as mean ± 1 S.D.). The TKN concentration in the control boxes was 1870 ±47 mg NH_3_-N kg^−1^ (the initial soil contained 1900 ±54 mg NH_3_-N kg^−1^), and this increased to 2120 ±131 and 2400 ±185 mg NH_3_-N kg^−1^ with the typical and high biosolid application rates, respectively. The SOM content of the control boxes was 2.8% ±0.1% (the initial soil also contained 2.8% ± 0.1%), and this increased to 3.0% ±0.1% and 3.3% ±0.2% with the typical and high biosolid application rates, respectively. Soil pH decreased slightly with the amount of biosolids applied, from an average value of 7.6 ±0.1 in the control test (7.4 ±<0.1 in initial soil) to 7.5 ±0.1 and 7.3 ±0.1 with the typical and high-rate tests.

**Table 1:** Soil properties (total Kjeldahl nitrogen, plant available P, soil organic matter content, and pH) of the initial soil and samples from the growth boxes. The mean values are reported ± the standard deviation

	Sampling stage	Initial soil			Box biosolid application																	
					Control (0 t/ha)						Typical (24 t/ha)						High (48 t/ha)					
					Replicate 1			Replicate 2			Replicate 1			Replicate 2			Replicate 1			Replicate 2		
TKN (mg NH_3_-N kg^−1^)	Pre-flood	1903	±	54	1836	±	30	1859	±	30	1995	±	150	2187	±	176	2382	±	155	2466	±	112
	Post-flood				1854	±	31	1913	±	35	2213	±	74	2098	±	19	2384	±	329	2358	±	133
	Final				1901	±	19	1879	±	66	2053	±	26	2178	±	71	2460	±	113	2324	±	122
Olsen P (mg P kg^−1^)	Pre-flood	19.83	±	3.34	31.88	±	0.94	25.04	±	1.36	35.70	±	2.74	38.08	±	3.58	41.60	±	4.14	41.35	±	3.15
	Post-flood				31.35	±	1.67	27.18	±	1.75	41.20	±	1.48	40.57	±	1.48	40.22	±	6.70	38.38	±	2.18
	Final				28.52	±	1.03	22.20	±	1.90	35.96	±	2.72	41.11	±	2.34	42.68	±	2.97	35.40	±	5.12
SOM (%)	Pre-flood	2.82	±	0.09	2.88	±	0.04	2.77	±	0.06	3.07	±	0.15	3.02	±	0.08	3.44	±	0.19	3.42	±	0.04
	Post-flood				2.83	±	0.03	2.85	±	0.04	3.18	±	0.19	3.02	±	0.04	3.22	±	0.31	3.26	±	0.10
	Final				2.80	±	0.06	2.71	±	0.01	2.99	±	0.02	2.95	±	0.05	3.41	±	0.09	3.18	±	0.10
pH	Pre-flood	7.40	±	0.05	7.64	±	0.00	7.63	±	0.05	7.48	±	0.05	7.45	±	0.05	7.31	±	0.05	7.26	±	0.00
	Post-flood				7.56	±	0.05	7.50	±	0.05	7.33	±	0.05	7.47	±	0.00	7.30	±	0.10	7.32	±	0.00
	Final				7.72	±	0.05	7.72	±	0.05	7.53	±	0.05	7.57	±	0.00	7.29	±	0.05	7.44	±	0.00

### Floodwater environmental monitoring

Surface water remained oxygenated during the flood event in all mesocosms, with a DO concentration of 8.46 ±0.90 mg O_2_ L^−1^. Porewater DO concentrations measured at 5cm and 10cm depth fell to >0.20 mg O_2_ L^−1^ within 6 h of flooding and to 0 mg O_2_ L^−1^ within 12 h, with no trend observed based on biosolid application rate. Surface water ORP, like DO, remained high (529 ±24 mV) during the experiment with no trend based on biosolid application rate. Porewater ORP at 5cm and 10cm depths was 445 ±45 mV 3 h after inundation. After 96 h, the porewater ORP had dropped to 217 ±11 mV in most boxes and then continued to drop steadily during the experiment to 192 ±13 mV before flood draining. The exception was the high-rate test (1) in which ORP continued to drop faster than other boxes after 96 h to 141 mV before flood draining.

### Bacterial community composition and diversity

#### Sample DNA yield

DNA yield from each sample was estimated from the extract concentration, final extraction volume, and initial sample weight (the maximum DNA recovery possible with the DNeasy PowerSoil Kit and the QIAquick gel extraction kit are 20× and 10× the highest DNA recovery from any sample, respectively). Biosolid samples yielded an order of magnitude more DNA (237 ± 55 μg g^−1^) than the initial soil samples (17 ± 10 μg g^−1^). Estimated DNA yield from the soil in the test boxes showed a lot of scatter, but no temporal trend. However, the high-rate boxes yielded more DNA (51 ± 21 μg g^−1^) than either the typical rate (28 ± 15 μg g^−1^) or control boxes (29 ± 17 μg g^−1^).

#### OTU clustering and diversity

Illumina MiSeq sequencing produced a total of 10.7M paired end reads, of which >96% passed quality control and filtering, and >88% were successfully mapped to an OTU. A further 2.1% of sequences were excluded from further analysis either because they were classified as Archaea or not classified to a bacterial phylum with a confidence of at least 0.7. Between 39,738 and 324,696 sequences per sample were mapped to a total of 4574 OTUs (mean: 145,416 reads per sample).

The biosolid samples contained sequences from a total of 527 OTUs, with the top 3 most abundant OTUs accounting for ~25% of all sequences, and the 13 most abundant OTUs contained >50% of total sequences. The initial soil samples contained sequences from all 4574 OTUs in the analysis, whereas the mesocosm soil samples contained sequences from 4467 OTUs (the 107 OTUs present in the initial soil but absent from the mesocosm soils together contain only 0.01% of total sequences). The 20 most abundant OTUs in the soils accounted for >20% of sequences, and >50% of total sequences were contained within the top 136 most abundant OTUs.

OTUs were classified into 38 different bacterial phyla (the biosolids contained sequences classified to 27 different phyla, and the mesocosm soils contained representatives from all 38 phyla). Alpha diversity measured using Hill numbers showed that the biosolid samples had a much lower diversity than either the initial or mesocosm soil samples (which had similar diversity; S.I. Figures S1, S2, S3). The biosolids had mean diversity indices of *D*_0_ = 296 ± 35, *D*_1_ = 62.7 ± 1.5, *D*_2_ = 34.79 ± 1.27, whereas the soil samples had mean diversity indices of *D*_0_ = 2844 ± 280, *D*_1_ = 658.8 ± 55.6, and *D*_2_ = 219.69 ± 28.53 (where D_0_, D_1_, and D_0_ are measures of the number of species, number of common species, and number of dominant species). Furthermore, there were only very small differences in diversity measures at D_0_, D_1_, or D_2_ between any soil sample regardless of biosolid application or flooding.

#### Taxonomic composition

All soil samples contained a taxonomically similar range of OTUs (pairwise Bray-Curtis dissimilarity scores were 0.396 ± 0.136, S.I. Table S2), although there was more dissimilarity between the initial soil samples and soil samples from the mesocosms, than between the mesocosm samples (Bray-Curtis dissimilarity scores 0.438 ± 0.105 and 0.389 ± 0.139, respectively). However, all soil samples were highly dissimilar to biosolid samples (pairwise Bray-Curtis dissimilarity score 0.984 ± 0.016). Dissimilarity was greatest between the biosolids and the control tests (Bray-Curtis dissimilarity score 0.999 ± 0.001) but still high in the tests with typical and high biosolid application rates (Bray-Curtis dissimilarity scores 0.978 ± 0.017 and 0.975 ± 0.014, respectively), indicating that the soil taxonomy was largely unchanged by the application of biosolids.

**Fig. 1:**
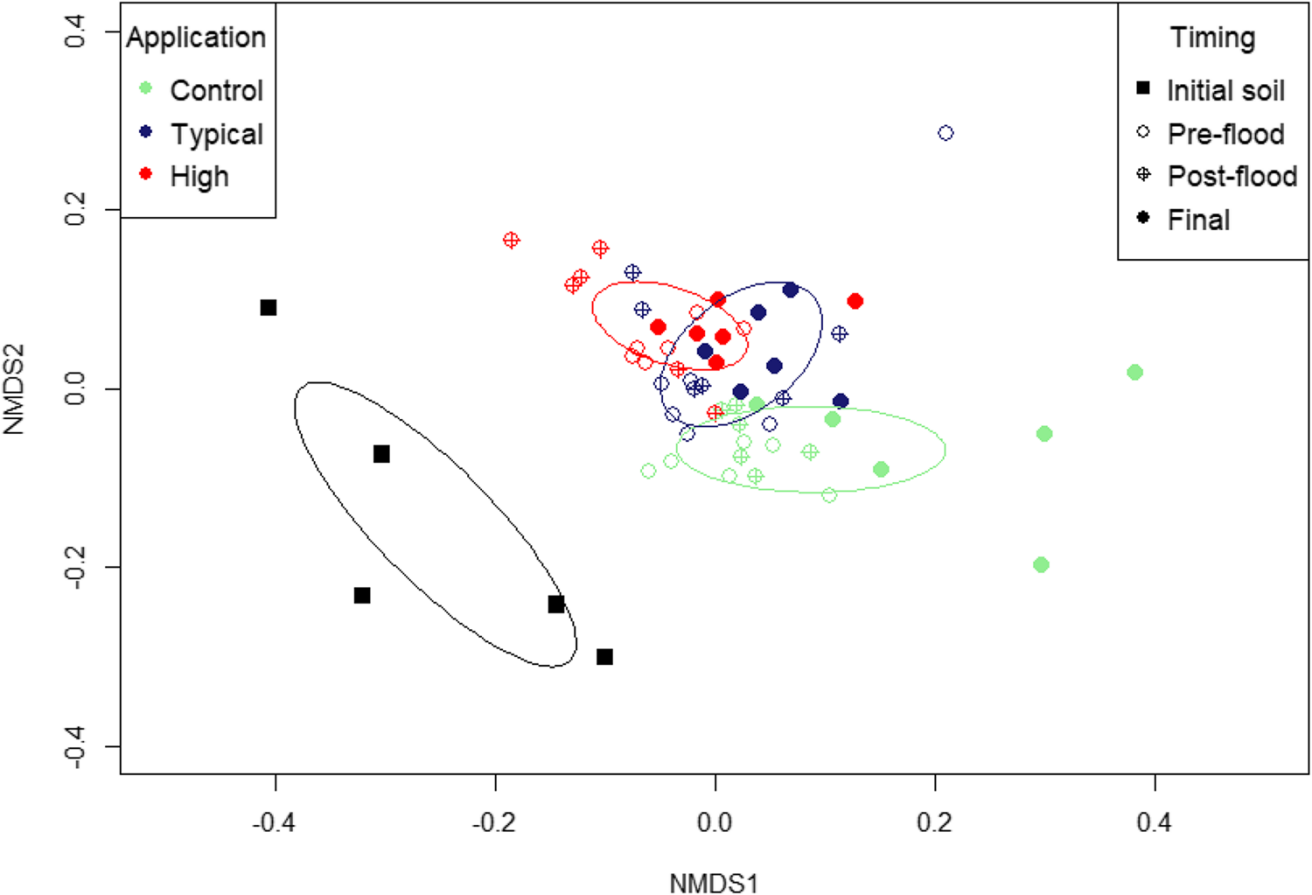
Non-metric multidimensional scaling (NMDS) plot showing dissimilarities between soil bacterial community samples determined by Bray-Curtis distances (*k* = 2, stress = 0.162). Points are coloured to represent biosolid application to boxes, and shape fills represent the different sample timings of initial soils, pre-flood, post-flood, and final. Ellipses represent the standard deviation of the points belonging to initial soils and biosolid applied soils at all timings and are coloured according to application

The modest dissimilarity between the initial soil and mesocosm soils is illustrated in the NMDS plot (Fig. 1: the biosolid samples are not shown as including such a dissimilar population would mask the differences within the soil populations). The initial soil samples group separately from the mesocosm soils, which form a broad group despite differences in biosolid application rate. This indicates that preparations from the growth experiments and crop growth influenced the microbial populations. Samples from the high-rate tests plotted in a region of the NMDS plot that overlaps with the typical-rate test samples, which in turn overlap with the region where samples from the control tests plot, but there is little overlap between the high rate and control groups.

**Fig. 2:**
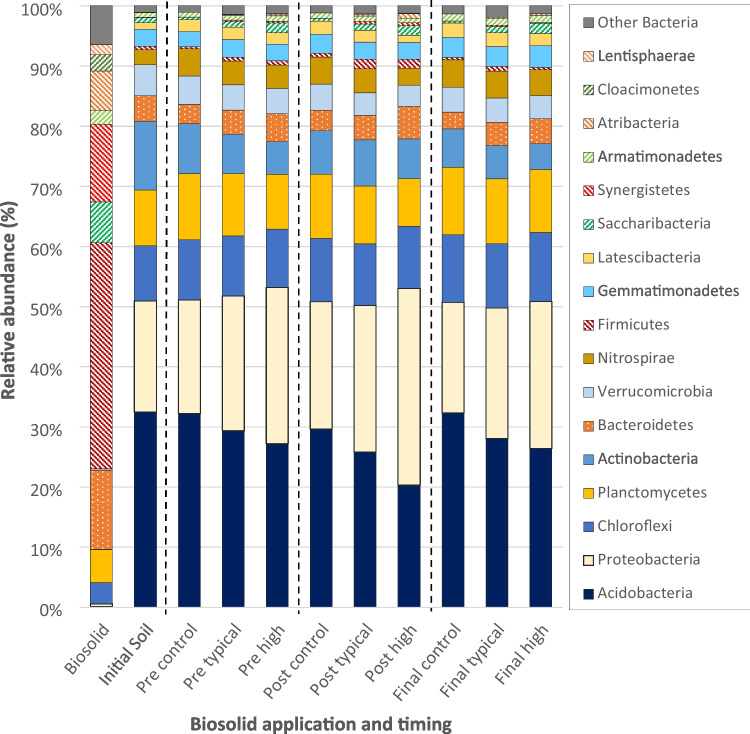
Average taxonomical composition of samples. Biosolid and initial soil are the mean results from the five samples of each material. Timing and biosolid application rate results represent the mean of the six samples from both boxes treated at each rate from each sampling timing. Taxonomies are ordered bottom to top by mean abundance across all samples. Taxonomies with <1% abundance in any sample after averaging are grouped with ‘Other Bacteria’ alongside unassigned phyla

About half of all sequence reads from the soil samples were assigned to two bacterial phyla (Fig. 2, S.I. Tables S3-S6); *Acidobacteria* (relative abundance (RA) 28.3 ± 4.4%) and *Proteobacteria* (22.9 ± 4.9%), which were barely present in biosolids (0.2 ± <0.1% and 0.4 ± <0.1%, respectively). *Chloroflexi* (10.3 ± 1.3%), *Planctomycetes* (10.0 ± 1.2 %), and *Actinobacteria* (6.9 ± 2.5 %) were the next most abundant phyla in the soil samples, and nearly 80% of reads from the soil samples were assigned to these five most abundant phyla. In comparison, nearly 80% of reads from the biosolids were assigned to the phyla *Firmicutes* (37.6 ± 0.7%), *Bacteroidetes* (13.2 ± 0. 7%), *Synergistetes* (12.9 ± 0.9%), *Saccharibacteria* (6.8 ± 1.2%), and *Atribacteria* (6.6 ± 0.3%), which had low RA in the soil samples (combined RA typically 6.1%, of which 4.0% were *Bacteroidetes*).

#### Microbial phylum correlations with soil properties

In the soil samples, the RAs of the phyla *Proteobacteria*, *Bacteroidetes*, *Saccharibacteria*, *Synergistetes*, *Atribacteria*, *Cloacimonetes*, and *Lentisphaerae* exhibit a significant positive correlation (*p*<0.001) with TKN, Olsen P, and SOM content, and a significant negative correlation (*p*<0.01) with pH (Table 2). Conversely, the RA of *Acidobacteria* exhibits a significant (*p*<0.001) negative correlation with TKN, Olsen P, and SOM content and a significant (*p*<0.01) positive correlation with pH. Soil TKN, Olsen P, and SOM all increased, and the pH decreased, with the biosolid application rate, suggesting that these populations responded to the application of biosolids. As the RA of *Proteobacteria* was very low in biosolid samples, and no taxa were introduced to the soil by the biosolids, the increase in RA of *Proteobacteria* is because the addition of the biosolids caused a shift in the existing soil bacterial community. Similarly, the decrease in RA of *Acidobacteria* must also reflect the impact of the biosolids addition on the existing soil bacterial community.

**Table 2:** Spearman’s rank correlations between RAs of soil bacterial phyla and soil properties. Phyla are in order of relative abundance across all soil and biosolid samples. An asterisk (*) indicates a significant correlation at *p*<0.05, a double asterisk (**) indicates a significant correlation at *p*<0.01, a triple asterisk (***) indicates a significant correlation <0.001

Bacterial phylum	TKN	Olsen P	SOM	pH
Acidobacteria	−0.730***	−0.767***	−0.723***	0.656**
Proteobacteria	0.802***	0.821***	0.858***	−0.781***
Chloroflexi	−0.018	−0.096	−0.140	0.225
Planctomycetes	−0.565*	−0.439	−0.700**	0.774***
Actinobacteria	−0.407	−0.381	−0.381	0.119
Bacteroidetes	0.811***	0.725***	0.825***	−0.846***
Verrucomicrobia	−0.565*	−0.502*	−0.519*	0.391
Nitrospirae	−0.401	−0.248	−0.381	0.573*
Firmicutes	0.418	0.518*	0.425	−0.449
Gemmatimonadetes	−0.019	−0.123	−0.123	0.267
Latescibacteria	−0.372	−0.279	−0.456	0.647**
Saccharibacteria	0.849***	0.716***	0.879***	−0.823***
Synergistetes	0.721***	0.714***	0.728***	−0.649**
Armatimonadetes	0.005	0.025	−0.070	0.284
Atribacteria	0.860***	0.746***	0.842***	−0.818***
Hydrogenedentes	0.318	0.295	0.168	0.063
Cloacimonetes	0.795***	0.735***	0.775***	−0.768***
Cyanobacteria	0.114	0.105	0.142	−0.168
Lentisphaerae	0.709***	0.663**	0.782***	−0.740***

The RA of *Bacteroidetes* taxa in the biosolids samples was 13.2 ±0.7% compared with 4.3 ±0.8% in the initial soil samples. While it was within error of the initial soil samples, there was also a pattern of increasing RA of *Bacteroidetes* taxa with biosolid application rate (the RAs were 3.1 ±0.4%, 4.0 ±0.8%, and 4.6 ±1.1%, respectively, in the control, typical, and high application rate tests). Of the 14 *Bacteroidetes* taxa that individually exceeded 0.1% mean RA in the biosolids, three had a higher RA in the biosolid-amended tests than in either the control tests or initial soil and a RA that increased with the rate of amendment (two were in the same class as *Vadin HA17*, and one was a genus of *Rikenellaceae*). Together these taxa had a RA of <0.04% in both the initial soil and control tests but RAs of 0.30% and 0.50% in the typical- and high-rate tests (their collective RA was 5.32% in the biosolids). The RA of these increased after flooding but decreased again during the final recovery period.

The RA of *Saccharibacteria* taxa in the biosolids samples was 6.8 ±1.4% compared to a mean RA of 0.9 ±0.2% in the initial soil samples. There was a pattern of increasing RA of *Saccharibacteria* taxa with biosolid application rate, with RAs of 0.4 ±0.1%, 1.0 ±0.4%, and 1.6 ±0.6%, respectively, in the control, typical, and high application rate tests.

The phyla *Synergistetes* and *Atribacteria* were abundant in the biosolid samples (RAs 12.9 ±1.1% and 6.6 ±0.4%, respectively) but scarce in the initial soil samples (RAs 0.06 ±0.05% and 0.04 ±0.04%, respectively). Like *Saccharibacteria*, both were more abundant in the tests where biosolids were applied than in either the control tests or initial soil (the RAs of *Synergistetes* were <0.01 ±<0.01%, 0.3 ±0.3%, and 0.3 ±0.2%. The *Atribacteria* RAs were <0.01 ±<0.01%, 0.2 ±0.2%, and 0.4 ±0.4%, respectively, in the control, typical, and high application rate tests). Importantly, in the tests where biosolids were applied, the RA of both phyla doubled during flooding, before returning to their pre-flood value after the recovery period. All the *Synergistetes* and *Atribacteria* taxa demonstrated similar patterns in RA with the biosolid application rate and flooding. *Cloacimonetes* and *Lentisphaerae*, the remaining phyla where RA exhibited a significant correlation with TKN, Olsen P and SOM content, displayed similar trends to *Synergistetes* and *Atribacteria.* However, as their RAs never exceed 0.1% in any soil sample, detailed patterns are difficult to discern.

*Firmicutes* was the dominant phylum in the biosolids (RA 37.6 ± 0.7%) but had a low abundance in soils, where its RA was not sensitive to the application of biosolids (pre-flood the RA was 0.7 ± 0.2% where biosolid was applied and 0.3 ± 0.2% in the control tests, which are comparable with 0.6 ± 0.5% in the initial soil). However, the RA of *Firmicutes* doubled during flooding (to 1.5 ± 0.7% where biosolids were applied and 0.6 ± 0.3% in the control tests), before returning to the pre-flood values during the recovery period. The 15 most abundant *Firmicutes* taxa in the biosolids accounted for nearly three-quarters of the *Firmicutes* reads in the biosolids but less than one-tenth of *Firmicutes* reads in control tests. These same *Firmicutes* taxa accounted for half the *Firmicutes* reads in the biosolid-amended tests prior to flooding, 40% of *Firmicutes* reads after flooding, but only 25% of *Firmicutes* reads after the recovery period. Together, this indicates that the RA of *Firmicutes* taxa in the soil was sensitive to the flood event (increasing during the period of anoxia). Also, in the short term, that the application of biosolids may increase the RA of *Firmicutes* taxa due to transfer from the biosolids, but that the second effect diminishes with time.

## Discussion

### Changes in diversity of soil bacteria during the experiment

The first notable finding of this study is that biosolids from AD of sewage sludge did not introduce new bacterial taxa into an arable soil. All the bacterial taxa in the biosolids were also present in the initial soil samples although at far lower abundances than in the biosolids (the D_1_ common species of the biosolids had a RA of 88.1 ±1.0% in the biosolids and 0.5 ±0.5% in the initial soil). This may simply reflect the diversity of bacteria found in soil or may be associated with land management practices at the farm (e.g. use of manures).

The Hill numbers (D_2_, D_1_, and D_0_) indicate that the initial soil and all the mesocosm soils had a similar bacterial diversity, despite the application of biosolids to some mesocosm soils. Conversely, the NMDS analysis (Fig. 1) indicates that there were modest differences between the mesocosm soils depending on biosolid application rate and a slightly larger difference to the initial soil. The NMDS analysis was based on the Bray-Curtis scores which give equal weighting to all taxa, so relatively low abundance taxa can have a disproportional effect on the analysis (107 OTUs with a combined RA of 0.10 ±0.03% in the initial soil were not detected in the mesocosm soils). However, the OTU table also showed a decrease in RA of several D_2_ and D_1_ bacterial species between the initial soil and controls. Many of these were taxa that were present in biosolid samples, suggesting they are well adapted to anaerobic conditions (they were classified to the phyla *Saccharibacteria*, *Bacteroidetes*, *Actinobacteria*, *Synergistetes*, and *Firmicutes*). More generally, the RA of the taxa that were common in the biosolids decreased between the initial soil and the start of the control tests (0.5% ±0.5% in the initial soil, but only 0.05 ±0.05% after 28 days of plant growth, decreasing to 0.01 ±0.01% at subsequent timepoints despite the flood event). The decrease in RA of these taxa in the control mesocosms suggests they are poorly adapted to the conditions of a well aerated soil.

DNA yields suggest that the biosolids contained approximately 10× more genetic material per gram than the initial soil. If the relationship between DNA recovery and bacterial numbers is the same for both materials, then the addition of biosolids will have initially increased bacterial numbers in the amended soils by around 8% and 15% for the typical and high application rates. Also, the biosolids had much lower diversity indices than soil samples (D_0_ and D_1_ were 10× smaller, and D_2_ was 6× smaller), so the dominant (D_2_) taxa in the biosolids were particularly abundant. However, despite the introduction of these highly abundant taxa, the bacterial populations of the biosolid-amended soils closely resembled control soils taxonomically and had similar diversity indices after 28 days of crop growth. Nonetheless, there were subtle differences in the biosolid-amended tests and the controls. The RA of the taxa that were common in the biosolids was 2.3 ±1.7% overall in the biosolid-amended tests (compare with 0.02% ±0.03% overall in the control tests), with the value increasing with application rate and during flooding (although the variations between application rates and timepoints are within error of the overall mean). Three of the D_2_ dominant taxa in the biosolids (*Saccharibacteria Candidatus Saccharimonas*, *Atribacteria Candidatus Caldatribacterium*, and *Bacteroidetes Vadin HA17*) were amongst the dominant species of mesocosm soil bacterial populations and showed trends of increasing RA with increasing biosolid application. These changes suggest that bacteria introduced into the soil by the biosolids persisted there over the 8 weeks of testing (possibly aided by the period of anoxia caused by flooding). Also, while the RA of the taxa introduced by the biosolids decreased during the flood recovery period to a value slightly lower than pre-flood, it is not known how long it would take for their RA to decrease to the level in the control soil.

### Impact of biosolids on soil geochemistry and bacterial populations

Eight bacterial phyla in the soil exhibited a significant correlation with four geochemical factors: TKN, Olsen P, SOM, and pH that changed following biosolid application (Table 2). However, these factors are not independent, as soil TKN, Olsen P, and SOM all increased with the biosolid application rate, and the pH decreased (probably due to increased respiration and nitrification processes in the soil). This means that the specific factors influencing the bacterial populations cannot be isolated, but significant correlation across all geochemical factors indicates bacterial phyla that were most influenced by biosolid application.

*Acidobacteria* and *Proteobacteria* were the dominant phyla in the soil regardless of biosolid application. These two phyla combined always accounted for between 49.78 and 53.22% of RA, with the RA of *Acidobacteria* decreasing and *Proteobacteria* increasing with biosolids. The RA of *Acidobacteria* showed a highly significant negative correlation with soil nutrients and a positive correlation with soil pH. *Proteobacteria* showed a highly significant positive correlation to soil nutrients and negative correlation with pH (Table 2). Members of both phyla are morphologically, physiologically, and metabolically diverse, but general trends correlate with phylum level classification (Gupta 2000; Fierer et al. 2007; Spain et al. 2009; Kielak et al. 2016). The decreased abundance of *Acidobacteria* with increased biosolids is consistent with previous studies which have found that *Acidobacteria* are more suited to soils with poor nutrient availability (Fierer et al. 2007; Ward et al. 2009; Sun et al. 2015; Kielak et al. 2016). Conversely, Proteobacteria have been shown to favour more nutrient-rich soils or soil rhizospheres where nutrients and SOM were more highly concentrated (Fierer et al. 2007; Sun et al. 2015; Zeng et al. 2016). This suggests that biosolid-applied soils were a more favourable environment for *Proteobacteria*, giving it a competitive advantage over the *Acidobacteria*.

The overall increase in RA of *Proteobacteria* does not appear to be related to the introduction of any new bacteria from the biosolid populations. *Proteobacteria* had a very low RA in biosolids, and the bacterial taxa present had very low RA in biosolid-applied test soils. The increased abundance of *Proteobacteria* therefore appears to reflect the response of native soil bacteria to nutrient additions from biosolids. The six most abundant *Proteobacteria* taxa (combined RA 4.1% in soil samples) belonged to the classes *β-Proteobacteria* and *γ-Proteobacteria.* These classes have been shown to have a positive relationship with SOM (Fierer et al. 2007; Li et al. 2017).

The RA of *Bacteroidetes* in the mesocosm soils was correlated with biosolid application rate, but the average RA was similar to that in the initial soil (where *Bacteroidetes* taxa were moderately abundant), and most of the variation was due to changes in the RA of taxa well-represented in native soil populations. *Bacteroidetes* are common in anaerobic digesters, as well as in gut microbiomes, but are also widely distributed in the environment (Miyashita 2015; Liu et al. 2016; Wang et al. 2019). They are linked to the breakdown of SOM and have been found to be positively correlated with soil organic C mineralisation rates (Fierer et al. 2007). Thus, it is speculated that the variation in RA with biosolid application rate is primarily a response to the addition of SOM from the biosolids.

Inspection of the OTU table revealed that most of the variation in the RA of phyla with biosolid application was due to changes in taxa that were already reasonably abundant in the soil. However, three *Bacteroidetes* classes exhibited RA patterns that suggest they were introduced with the biosolids (i.e. very low RA in the initial soil and control mesocosms, high RA in the biosolids, and elevated RA in the biosolid-amended tests). The two most abundant of these were classified as members of the uncharted family *Vadin HA1*, which are amino acid-degrading organisms found principally in anaerobic digesters, and recently named candidate *Aminobacteroidaceae* in the order *Bacteroidales* (Mei et al., 2020). Similarly, the high RA of *Saccharibacteria* in the biosolid samples and increase in their RA with biosolid application rate were both classified to the unconfirmed genus *Candidatus Saccharimonas.* The genome of *Candidatus Saccharimonas aalborgensis* was assembled from metagenomic analysis of activated sludge bioreactor samples and has been identified as an obligate anaerobe that ferments glucose and other sugars (Albertsen et al. 2013). The *Firmicutes* taxa that had elevated RA in the biosolid-amended tests relative to the control tests and initial soil were overwhelmingly classified as *Clostridiales*. This is an order of fermentative obligate anaerobes (Stackebrandt 2014), which may explain why their relative proportion of the phylum *Firmicutes* diminished over the test period, as obligate anaerobes are not well adapted to the soil environment. The *Synergistetes* taxa whose RA patterns in mesocosm soils suggest they were introduced with biosolids were classified to the family *Synergistaceae*, whose members are predominantly anaerobes, and several have been isolated from AD sludge (Soutschek et al. 1984; Baena et al. 1998; Díaz et al. 2007; Ganesan et al. 2008). The *Atribacteria* taxa which demonstrated patterns suggesting they may have been introduced with the biosolids were classified to the Candidate genus *Caldatribacterium*, an uncultivated bacterial lineage found in AD and similar habitats (Dodsworth et al. 2013).

Overall, the variation in soil bacterial populations with biosolid application rate appears to be due largely to the impact of nutrients and SOM on the native soil populations, with only a small number of taxa exhibiting a pattern which indicates their introduction with biosolids. Thus, over longer time frames, as increases in nutrients and SOM content from biosolid application are depleted, soil bacterial populations are likely to return to a status which is similar to the control soils.

### Effect of flooding on soil bacterial populations

Flooding did not have a dramatic effect on bacterial populations in the mesocosms. After 10 days of flooding, there were only modest differences in the RAs of the main bacterial phyla from those immediately before flooding, and the changes were largely reversed during the post-flood recovery period. The largest difference was that the RA of *Acidobacteria* decreased and *Proteobacteria* increased during the flood event. As a result, the mean RA of *Proteobacteria* exceeded that of *Acidobacteria* after flooding in the high biosolid application rate test (the only point where this occurred), due to the combined effect of biosolids application rate and flooding. This may reflect a greater diversity among the *Proteobacteria* than the *Acidobacteria*, making them more resilient to the change (Faoro et al. 2010; Miyashita 2015). Ten of the 15 most abundant *Proteobacteria* taxa in the analysis showed a >10% increase in RA during the flood; half were *β-Proteobacteria*, and half were *γ-Proteobacteria*. Both *β-Proteobacteria* and *γ-Proteobacteria* are phylogenetically and physiologically diverse, but many are facultative anaerobes, which makes them adaptable rhizosphere bacteria able to readily cope with periods of soil saturation (Marín 2014). Conversely, while *Acidobacteria* are abundant in soils and can compose half of the bacterial community in arid soils (Dunbar et al. 2002), they are predominantly aerobes (Eichorst et al. 2018), which may explain why they are negatively impacted by flooding. The high abundance of *Proteobacteria* and low abundance of *Acidobacteria* in many river sediments supports this supposition (Vidal Dura et al. 2018; Huang et al. 2019; Zhang et al. 2019).

The *Firmicutes*, the most abundant phylum in biosolids, were far less abundant in the mesocosm soils but nonetheless showed important changes. During the flood, the RA of the *Firmicutes* approximately doubled in all mesocosms (although from a lower baseline in the control test), before returning to the pre-flood value during the recovery period. *Firmicutes* are predominantly anaerobes (they are common in gut microbiomes, AD and anaerobic soils; (Sun et al. 2015; Liu et al. 2016; Jiang et al. 2019)), which probably accounts for their increased RA during flooding. Interestingly, the *Firmicutes* taxa that were abundant in the biosolids were found predominantly in biosolid-applied soils, and their proportionate representation within the *Firmicutes* diminished over time, suggesting that they were introduced with the biosolids and not as well adapted to soil environments as the native species. The two most abundant *Firmicutes* gena in biosolids were *Sedimentibacter* and *Syntrophomonas* (within the order *Clostridiales*), which have largely been isolated from strictly anaerobic environments (McInerney et al. 1981; Hatamoto et al. 2007; Imachi et al. 2016). In contrast, the three most abundant *Firmicutes* families in the mesocosm soils were native species, belonging to *Bacillaceae* and *Gracilibacteraceae*. The *Bacillaceae* taxon was one of the D_2_ dominant species in all soils, whereas the two *Gracilibacteraceae* taxa were among the D_2_ dominant species in the post-flood soil and the D_1_ common species in final soil but had very low abundance in pre-flood and initial soils. Very few members of the family *Gracilibacteraceae* have been fully characterised, but those which have are obligate anaerobes like most other members of the order *Clostridiales* (Gupta et al. 2018). However, the family *Bacillaceae* contains many facultative anaerobes (McBride and Turnbull 1998). Whatever their metabolism, it seems that the native *Firmicutes* species which are relatively dormant in the soil were able to capitalise on the period of anoxia induced by the flooding.

Members of the phyla *Synergistetes* and *Atribacteria* responded in a similar manner to the *Firmicutes*. However, their overall RA in the biosolid-amended mesocosms was a thousand times higher than in the control tests, and only increased during flooding in the biosolid-amended tests but not the controls. Thus, it is inferred that the increase upon flooding involved taxa introduced with the biosolids. Both *Synergistetes* and *Atribacteria* are associated with anaerobic environments (Jumas-Bilak and Marchandin 2014; Nobu et al. 2016), so were likely to flourish during the anaerobic flood conditions. After the 20-day flood recovery period, the RA of both phyla was similar to their pre-flood values, showing that flood-induced changes were relatively short-term. However, this behaviour differed from that of the *Firmicutes* taxa introduced with biosolids, whose RA decreased between the pre-flood and final timepoints. This suggests that *Synergistetes* and *Atribacteria* taxa introduced may remain in the soil for longer periods after biosolid application.

### Limitations and further work

This paper evaluated changes to the soil microbial population due to biosolid use, but it should be noted that not all biosolid-introduced bacteria will have negative effects on the soil microbiome. Indeed, several studies have shown that biosolid application can enhance soil microbial diversity and community function by increasing genes beneficial to plant growth and disease suppression (Curci et al. 2020; Stavridou et al. 2021). As discussed, biosolid application did have a small impact on soil bacterial diversity in comparison with the control soil. However, as this study did not investigate changes in the genetic capability of the bacterial populations, it is unknown whether the observed changes in diversity and the RAs of different bacterial phyla will have a beneficial or detrimental effect on soil function in the long term.

The mesocosm experiments in this study were designed to simulate flooding of a typical arable soil, inclusive of vegetation growth, as closely as practically possible in a laboratory setting. While this allowed for more controlled conditions for detailed monitoring of changes to the soil microbiome, there are limitations in what can be achieved at lab scale. As with all carefully controlled mesocosm studies, there is need to validate any findings at field scale. Also, while this study answered its intended research question, more research is needed to understand how these findings might translate to a wider range of soil properties, environmental variations, and soil management practices. Further work is needed to verify if biosolids from different sources behave in similar ways (e.g. those from different water treatment works and different AD systems without thermal hydrolysis pretreatment). Investigation is also needed to assess the rate of post-flood recovery of the soil microbiome from longer-term flooding where the soils remain anaerobic for extended periods.

## Conclusions

Application of biosolids to an arable soil at rates which reflect UK agricultural practice caused only modest changes in soil bacterial populations. No new species were introduced to arable soils by biosolid application, although the RAs of certain native species were increased. The main differences which were observed between the bacterial populations of the biosolid-applied and control tests were largely due to the impact of nutrients and SOM on the native soil populations. The principal change from biosolid application was that the RA of native soil taxa classified as *Proteobacteria* increased, whereas *Acidobacteria* decreased.

Flooding of soils induced changes in soil bacterial populations that probably reflect the soil becoming anaerobic (pore water DO was below detection limit). Again, the principal change from flooding was that the RA of the taxa *Proteobacteria* increased and *Acidobacteria* decreased, compounding the changes observed from biosolid application. The RA of *Firmicutes* taxa also roughly doubled after flooding, although from a far lower initial value. After flooding ceased, the soil bacterial populations recovered to their pre-flood profiles, indicating that any shifts were a transient response to the influx of bacteria from the biosolids and temporal change in soil ORP conditions induced by flooding.

Despite some small transference of bacterial species from biosolids to soils, the native soil microbiome appears to be highly robust and remain largely unaffected. However, under flood conditions, there is a risk that some anaerobic bacteria introduced in trace amounts to soil from biosolids could increase in abundance. The precautionary principle would therefore suggest that the application of biosolids to land at risk of flooding should be avoided if there are concerns about the sensitivity of the wider environment. However, it is unknown if these bacteria are harmful to the environment, and may even be beneficial, and upon alleviation of flood conditions, the soil microbiome recovered quickly to a pre-flood state. Given proper management of arable soils, and application rates within the currently recommended best practice, biosolids appear to be a safe and valuable nutrient source for crops and the soil microbiome.

## Data Availability

Data is available on request from the corresponding author.

## Supporting information

ESM 1: Additional supporting information may be found online in the Supporting Information attachment for this article.
