# Supplementary material for: Response of soil bacterial populations to application of biosolids under short-term flooding
Source: Environ Sci Pollut Res Int. 2023 May 15;30(28):72978–92. doi: 10.1007/s11356-023-27424-0 (PMC10257635; doi:10.1007/s11356-023-27424-0)
Supplement: ESM 1: — Additional supporting information may be found online in the Supporting Information attachment for this article. [file 11356_2023_27424_MOESM1_ESM.docx]

# **Supporting Information**

## Response of soil bacterial populations to application of biosolids under short-term flooding

Nicholas H. Humphries^1,2^*, Steven F. Thornton^3^, Xiaohui Chen^1^, Andrew W. Bray^4,5^,

Douglas I. Stewart^1^

^1^ School of Civil Engineering, University of Leeds, Leeds, LS2 9JT, UK

^2^ Currently Anglo American plc, 17 Charterhouse St, London, EC1N 6RA, UK

^3^ Department of Civil and Structural Engineering, University of Sheffield, Sheffield, S1 3JD, UK

^4^ School of Earth and Environment, University of Leeds, Leeds, LS2 9JT, UK

^5^ Currently Calder Rivers Trust, Walton, Wakefield, WF2 6TF, UK

* **Corresponding author**: N.H. Humphries, +447981 959537, [n.humphries504@gmail.com](mailto:n.humphries504@gmail.com)

The work reported in this paper was untaken by the first author in partial fulfilment of the requirements for the degree of Doctor of Philosophy. More details are available open access at: <https://etheses.whiterose.ac.uk/28068/>

Table S1 – Biosolid nutrient analysis results.

| Factor | Units | Value | Factor | Unit | Value |
| --- | --- | --- | --- | --- | --- |
| pH |  | 8.25 | Zn | mg/kg | 631 |
| DM | % | 26.4 | Na | mg/kg | 797 |
| Total N | % w/w | 5.39 | Ca | mg/kg | 23934 |
| Ammonium N | mg/kg | 7984 | Fe | mg/kg | 47793 |
| Nitrate N | mg/kg | <10 | Mo | mg/kg | 7.91 |
| P | mg/kg | 29975 | Mn | mg/kg | 622 |
| K | mg/kg | 1270 | C | % w/w | 33.4 |
| Mg | mg/kg | 3405 | Co | mg/kg | 11.5 |
| S | mg/kg | 13004 | B | mg/kg | 11.8 |
| Cu | mg/kg | 213 | C:N Ratio |  | 6.20 |

Figure S1 – Richness (D_0_) of species within samples measured using Hill Numbers. Error bars indicate standard deviation. Biosolid and initial soil results are based on the five (5) samples of each material. Box applications are based on six (6) samples from both boxes containing the respective biosolid application (control (0 t/ha), typical (24 t/ha, high (48 t/ha)) and at the stated sample timing (pre-flood, post-flood or final).

Figure S2 – Common species (D_1_) within samples measured using Hill Numbers. Error bars indicate standard deviation. Biosolid and initial soil results are based on the five (5) samples of each material. Box applications are based on six (6) samples from both boxes containing the respective biosolid application (control (0 t/ha), typical (24 t/ha, high (48 t/ha)) and at the stated sample timing (pre-flood, post-flood or final).

Figure S3 – Dominant species (D_1_) within samples measured using Hill Numbers. Error bars indicate standard deviation. Biosolid and initial soil results are based on the five (5) samples of each material. Box applications are based on six (6) samples from both boxes containing the respective biosolid application (control (0 t/ha), typical (24 t/ha, high (48 t/ha)) and at the stated sample timing (pre-flood, post-flood or final).

*Table S2 - Average Bray-Curtis dissimilarity scores. Results displayed are the mean scores for the comparison of samples within one group with each sample in a second group. A score of 1 indicates that samples do not share any species and a score of 0 indicates the samples are the same. The diagonal therefore represents the comparison of all samples within its own group, including the 0 score for each individual sample’s comparison with itself. The table is colour coded with red showing more dissimilarity and green indicating less dissimilarity between samples.*

|  | **Biosolid** | **Initial** | **Pre 0g** | **Post 0g** | **Final 0g** | **Pre 400g** | **Post 400g** | **Final 400g** | **Pre 800g** | **Post 800g** | **Final 800g** |
| --- | --- | --- | --- | --- | --- | --- | --- | --- | --- | --- | --- |
| **Biosolid** | 0.196 | 0.992 | 0.999 | 0.998 | 0.999 | 0.983 | 0.970 | 0.979 | 0.975 | 0.966 | 0.985 |
| **Initial** | 0.992 | 0.330 | 0.388 | 0.395 | 0.494 | 0.375 | 0.457 | 0.443 | 0.417 | 0.516 | 0.457 |
| **Pre 0g** | 0.999 | 0.388 | 0.276 | 0.342 | 0.453 | 0.315 | 0.420 | 0.370 | 0.378 | 0.503 | 0.397 |
| **Post 0g** | 0.998 | 0.395 | 0.342 | 0.230 | 0.405 | 0.294 | 0.370 | 0.364 | 0.339 | 0.462 | 0.365 |
| **Final 0g** | 0.999 | 0.494 | 0.453 | 0.405 | 0.406 | 0.434 | 0.461 | 0.453 | 0.451 | 0.526 | 0.460 |
| **Pre 400g** | 0.983 | 0.375 | 0.315 | 0.294 | 0.434 | 0.213 | 0.365 | 0.341 | 0.298 | 0.440 | 0.341 |
| **Post 400g** | 0.970 | 0.457 | 0.420 | 0.370 | 0.461 | 0.365 | 0.366 | 0.403 | 0.376 | 0.445 | 0.405 |
| **Final 400g** | 0.979 | 0.443 | 0.370 | 0.364 | 0.453 | 0.341 | 0.403 | 0.315 | 0.372 | 0.462 | 0.367 |
| **Pre 800g** | 0.975 | 0.417 | 0.378 | 0.339 | 0.451 | 0.298 | 0.376 | 0.372 | 0.255 | 0.407 | 0.339 |
| **Post 800g** | 0.966 | 0.516 | 0.503 | 0.462 | 0.526 | 0.440 | 0.445 | 0.462 | 0.407 | 0.384 | 0.443 |
| **Final 800g** | 0.985 | 0.457 | 0.397 | 0.365 | 0.460 | 0.341 | 0.405 | 0.367 | 0.339 | 0.443 | 0.311 |

*Table S3 - Bacterial phyla relative abundances (%) in biosolid and initial soil samples. Phyla marked with and asterisk (*) were included as 'Other bacteria' in the main analysis.*

*Table S4 - Pre-Flood soil sample bacterial phyla relative abundances (%). Phyla marked with and asterisk (*) were included as 'Other bacteria' in the main analysis.*

*Table S5 - Post-Flood soil samples bacterial phyla relative abundances (%). Phyla marked with and asterisk (*) were included as 'Other bacteria' in the main analysis.*

*Table S6 - Final soil samples bacterial phyla relative abundances (%). Phyla marked with and asterisk (*) were included as 'Other bacteria' in the main analysis.*
